# Association between depression and mortality in persons with asthma: a population-based cohort study

**DOI:** 10.1186/s13223-022-00672-4

**Published:** 2022-04-01

**Authors:** Ping Lin, Xiaoqian Li, Zongan Liang, Ting Wang

**Affiliations:** Department of Respiratory and Critical Care Medicine, West China School of Medicine and West China Hospital, Sichuan University, Chengdu, 610041 China

**Keywords:** Asthma, Depression, NHANES, Mortality, US adults

## Abstract

**Background:**

The relation between depression and mortality in patients with asthma is not well known. This study aimed to assess the impact of depression on mortality in asthmatic patients in US adults.

**Methods:**

This observational study used data from the 2005 to 2014 National Health and Nutrition Examination Survey (NHANES). Depression was measured using the Patient Health Questionnaire-9 (PHQ-9). We used survey-weight adjusted Cox proportional hazard models to estimate hazard ratios (HRs) with 95% confidence intervals (CIs) for the association between depression and all-cause mortality.

**Results:**

A total of 1865 participants with asthma were included in this study. Among them, 264 (14.16%) had depressive symptoms. During 9970 person-years of follow-up, there were 24 (9.1%) deaths in 264 patients with depression compared with 100 (6.3%) deaths in 1601 patients without depression. For unadjusted analyses, depression was associated with an increased risk of all-cause mortality (HR, 2.22 [95% CI 1.32–3.73]). This association was persistent after adjustment for age, sex, race/ethnicity, and body mass index (HR, 2.71 [95% CI 1.58–4.66]). However, we did not observe a significant association between depression and mortality after controlling for extensive co-morbidities (HR, 1.92 [95% CI 0.82–4.45]). Subgroup analyses further revealed that depression was an independent risk factor for mortality only in the females (HR, 3.78 [95% CI 1.17, 12.26]) but not all asthmatic patients.

**Conclusions:**

The present study suggested that depressive disorder was common in asthmatic patients and depression in asthmatic patients was associated with a higher mortality rate. Depression was an independent risk factor for mortality in female patients.

## Introduction

Asthma is a common chronic inflammatory disease of the airways, characterized by bronchial hyper-responsiveness and reversible airway obstruction, resulting in a substantial worldwide burden of disease [1, 2]. The overall prevalence of asthma is increasing worldwide, especially among women and children [3]. Growing evidence indicates that patients with asthma often suffer from depression and anxiety, with an estimated prevalence of about 10% [4]. The mechanisms that might link asthma and major depressive disorder were complex, involving autonomic nervous system dysregulation, early life stress, glucocorticoid resistance, and cytokine dysregulation [5]. Asthmatic patients with depression had less adherence to treatment of both asthma and comorbid diseases than those without depression and were more likely to experience an acute exacerbation [6].

Depression was associated with elevated mortality risk independently in the general population [7], and in patients with specific disorders such as chronic obstructive pulmonary disease [8], coronary heart disease [9], type 2 diabetes [10], and cancer [11]. Although depression was associated with poor asthma control, the impact of depression on long-term mortality in asthmatic patients was unknown [12]. Thus, in the present study, we aimed to fill this critical gap in knowledge by assessing the impact of depression on mortality in asthmatic patients in a nationally representative dataset from the National Health and Nutrition Examination Survey (NHANES). We hypothesized that asthmatic patients with depression would have increased mortality risk compared with those without depression.

## Methods

### Study population

NHANES, a national research survey aimed to assess the health and nutritional status of the civilian, non-institutionalized US population, was conducted by the National Center for Health Statistics (NCHS) of the Centers for Disease Control and Prevention (CDC) [13]. Standardized in-person interviews data and physical examinations results, including demographic, socioeconomic status, dietary, and health-related questions, were collected continuously (about 5000 persons each year) and released in 2 year cycles. Written informed consent was obtained from each participant before participation in this study.

For this study, we used survey data from the years 2005 to 2014. Participants were excluded from analyses if they were < 18 years, had incomplete data on baseline BMI and medical conditions, and had no asthma. The NCHS Public-Use Linked Mortality File through 31 December 2015 provided by the National Death Index (NDI) was used to ascertain death certificate information [14].

### Depression assessment

In NHANES, depression was measured using the Patient Health Questionnaire-9 (PHQ-9), a nine-item self-report instrument used as a screening and diagnostic tool [15]. Each instrument was given a point ranging from 0 to 3 according to the frequency of symptoms of depression over the past two weeks and the total PHQ-9 score ranges from 0 to 27. As done by previous studies [16], a PHQ-9 total score ≥ of 10 was regarded as clinically relevant depression in this study [17].

### Statistical methods

Baseline characteristics were described according to depression status. Means and standard deviations were used to describe continuous variables, whereas percentages were used for categorical variables. Continuous variables were analyzed using the weighted linear regression model, and categorical variables using the weighted chi-square test. We used Kaplan–Meier survival curves and Cox regression-based test for equality of survival curves to compare the overall survival of the patients with or without depression. We used survey-weight adjusted Cox proportional hazard models to estimate hazard ratios (HR) with 95% confidence intervals (CI) for the association between depression and all-cause mortality. Mode 1 was not adjusted. Model 2 was adjusted for age, sex, race/ethnicity (Mexican American, Other Hispanic, Non-Hispanic White, Non-Hispanic Black, Other Race), and body mass index (BMI). Model 3 was adjusted for age, sex, race/ethnicity, BMI, and extensive co-morbidities including diabetes, hypertension, congestive heart failure, stroke, coronary heart disease, chronic bronchitis, emphysema, cancer, asthma attack in the past year, and emergency care visit for asthma in the past year. Subgroup analyses were performed by examining age (< 60 years, ≥ 60 years), sex, and race/ethnicity. Statistical analyses were done using Stata version 14.0 (Stata Corp) and R version 3.6.3 (R Foundation for Statistical Computing) with appropriate sampling weights to account for the complex survey design. Statistical significance was defined as a two-tailed P value < 0.05.

## Results

### Participant characteristics

**Fig. 1:**
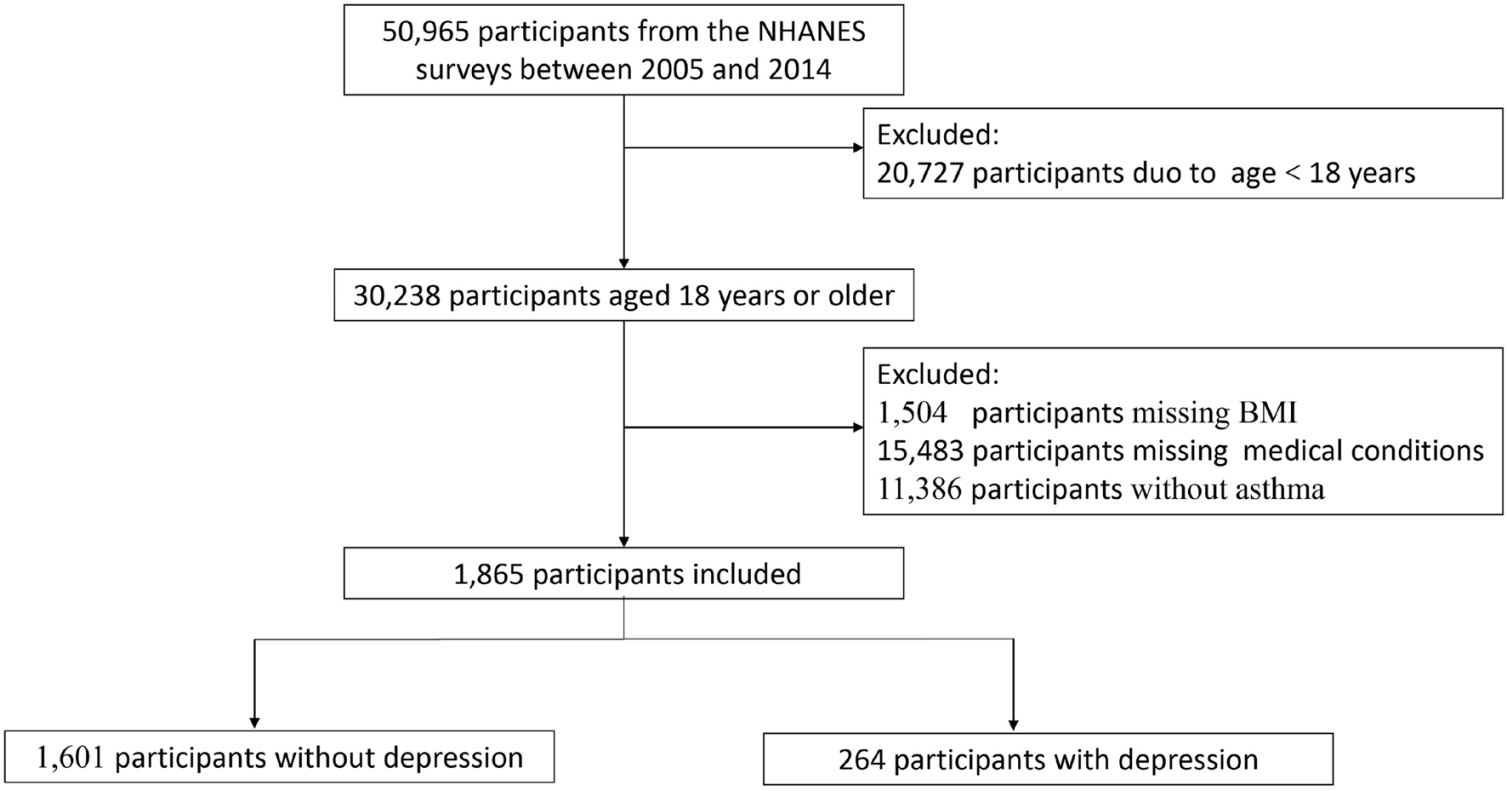
Flowchart of patient selection

**Fig. 2:**
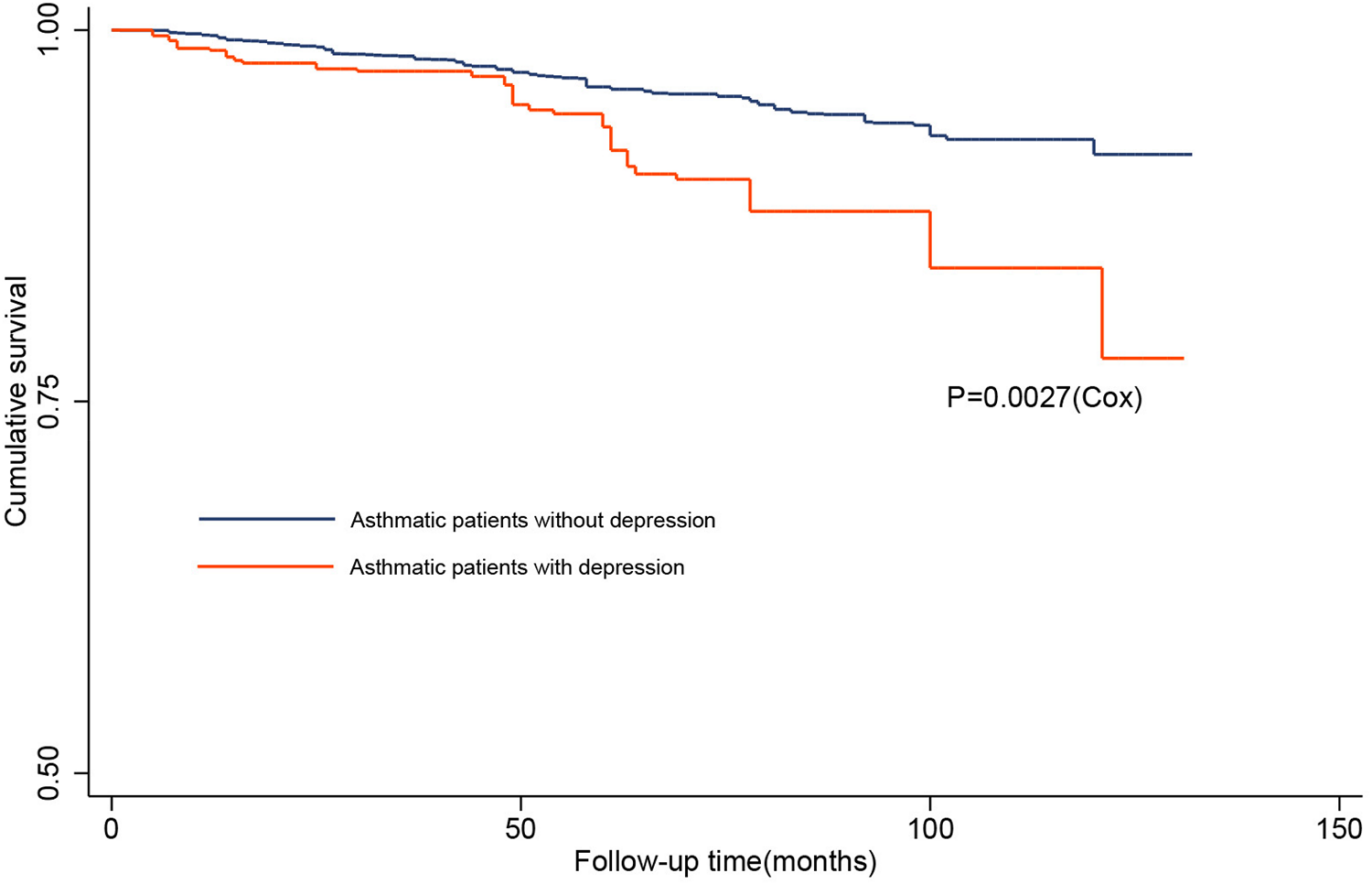
Kaplan–Meier curve depicts predicted overall survival

There were 50,965 participants from NHANES 2005 ~ 2014. Of these, 20,727 were < 18 years at the baseline survey, 1504 had no data on baseline BMI, 15,483 had incomplete data with respect to medical conditions, and 11,386 had no asthma, Thus, a total of 1865 participants were included in the current analysis (Fig. 1). The baseline characteristics of participants are presented in Table 1. Among 1865 individuals, 264 (14.16%) had depressive symptoms (PHQ-9 ≥ 10). Relative to asthma participants without depression, those with depression were more likely to be female (71.4% vs 55%), to have high BMI (33.3 ± 9.6 vs 29.8 ± 7.5), and to have hypertension (53.0% vs 33.8%), diabetes mellitus (18.5% vs 10.0%), coronary heart disease (9.4% vs 3.6%), congestive heart failure (8.2% vs 3.3%), stroke (8.4% vs 3.2%), emphysema (12.8% vs 3.9%), chronic bronchitis (39.3% vs 18.8%), cancer (15.6% vs 10.0%), asthma attack in past year (61.5% vs 49.6%), and emergency care visit for asthma in past year (28.2% vs 16.4%). During 9970 person-years of follow-up, there were 24 (9.1%) deaths in 264 patients with depression compared with 100 (6.3%) deaths in 1601 patients without depression. Kaplan–Meier curves demonstrated that asthmatic patients with depression were associated with an increased risk of all-cause mortality (p < 0.01) (Fig. 2).

**Table 1:** Patient baseline demographic and clinical characteristics

Characteristic	Without depression (N = 1601)	With depression (N = 264)	p value
Age (y)	44.6 ± 16.6	46.3 ± 14.1	0.158
Male (%)	45.0	28.6	< 0.001
BMI (kg/m2)	29.8 ± 7.5	33.3 ± 9.6	< 0.001
Race (%)			0.101
Mexican American	4.9	5.5	
Other hispanic	4.2	8.0	
Non-hispanic white	73.3	67.6	
Non-hispanic black	12.0	14.1	
Other races	5.6	4.7	
Hypertension (%)	33.8	53.0	< 0.001
Diabetes mellitus (%)	10.0	18.5	< 0.001
CHD (%)	3.6	9.4	< 0.001
CHF (%)	3.3	8.2	< 0.001
Stroke (%)	3.2	8.4	< 0.001
Emphysema (%)	3.9	12.8	< 0.001
Chronic bronchitis (%)	18.8	39.3	< 0.001
Cancer (%)	10.0	15.6	0.012
Asthma attack in past year (%)	49.6	61.5	0.007
EMC visit for asthma (%)	16.4	28.2	0.002

*BMI* body mass index, *CAD* coronary heart disease, *CHF* congestive heart failure, *EMC* emergency care

### Depression and all-cause mortality

Table 2 demonstrates associations of depression and total mortality, which were estimated by using unadjusted and adjusted models. For unadjusted analyses, depression was associated with an increased risk of all-cause mortality (HR, 2.22 [95% CI 1.32–3.73]). After controlling for age, sex, race/ethnicity, and BMI, depression remained a significant predictor of all-cause mortality (HR, 2.71 [95% CI 1.58–4.66]). However, we did not observe a significant association between depression and all-cause mortality after controlling for extensive co-morbidities (HR, 1.92 [95% CI 0.82–4.45]). In addition, the relationship between depression and mortality was related to the severity of depression before controlling for co-morbidities (Table 2). After controlling for co-morbidities, the relationship between depression and mortality was not related to the severity of depression.

**Table 2:** The association between depression and all-cause mortality in 1865 participants

Variables	Model 1 HR (95% CI)	p value	p for trend	Model 2 HR (95% CI)	p value	p for trend	Model 3 HR (95% CI)	p value	p for trend
All participants	**2.22 (1.32, 3.73)**	**0.003**		**2.71 (1.58, 4.66)**	** < 0.001**		**1.92 (0.82, 4.50)**	**0.135**	
PHQ-9 score									
≤ 10	**Reference**		**0.005**			** < 0.001**			**0.175**
10 < × < 15	**2.40 (1.27, 4.54)**	**0.007**		**2.94 (1.49, 5.80)**	**0.002**		**2.08 (0.73, 5.99)**	**0.171**	
≥ 15	**1.95 (0.89, 4.26)**	**0.093**		**2.38 (1.09, 5.21)**	**0.030**		**1.64 (0.52, 5.14)**	**0.394**	

Model 1 was unadjusted

Model 2 was adjusted for age, sex, race, and body mass index

Model 3 was adjusted for age, sex, race, body mass index, hypertension, diabetes, congestive heart failure, coronary heart disease, emphysema, stroke, chronic bronchitis, cancer, asthma attack in past year, and emergency care visit for asthma in past year

### Subgroup analyses

We further examined the association of depression with total mortality in different subgroups (Table 3). Multivariate model 3 showed that depression was independently associated with increased all-cause mortality among females (HR, 3.78 [95% CI 1.17, 12.26]), but this association in other subgroups was not significant.

**Table 3:** Subgroup analyses according to gender, age, race/ethnicity

Variables	Model 1 HR (95% CI)	p value	Model 2 HR (95% CI)	p value	Model 3 HR (95% CI)	p value
Subgroup analyses						
Gender						
Male	1.97 (0.88,4.42)	0.097	2.12 (0.92,4.89)	0.076	0.73 (0.17,3.13)	0.675
Female	2.36 (1.21,4.60)	0.012	3.05 (1.52,6.13)	0.002	3.78 (1.17,12.26)	0.027
Age						
Aged < 60 yeras	4.45 (2.06,9.61)	< 0.001	3.74 (1.67,8.29)	0.001	1.40 (0.29,6.90)	0.676
Aged ≥ 60 years	1.41 (0.64,3.11)	0.393	2.64 (1.27,5.51)	0.009	2.54 (0.23,28.41)	0.449
Race/ethnicity						
Mexican American	2.05 (0.36,11.60)	0.417	1.44 (0.19,10.81)	0.723	NA	
Other hispanic	0.33 (0.03,3.30)	0.347	0.41 (0.04,4.15)	0.454	NA	
Non-hispanic white	2.48 (1.32,4.66)	0.005	3.04 (1.58,5.87)	0.001	2.33 (0.72,7.52)	0.159
Non-hispanic black	1.94 (0.80,4.72)	0.143	2.57 (0.90,7.33)	0.077	4.94 (0.22,113.13)	0.317

Model 1 was unadjusted

Model 2 was adjusted for age, sex, race, and body mass index

Model 3 was adjusted for age, sex, race, body mass index, hypertension, diabetes, congestive heart failure, coronary heart disease, emphysema, stroke, chronic bronchitis, cancer, asthma attack in past year, and emergency care visit for asthma in past year

*NA* data was not available duo to limited sample size

## Discussion

In the present study, we found that depressive disorder was common in asthmatic patients and asthmatic patients with depression had a higher risk of all-cause mortality than those without depression. Furthermore, we found that depression was an independent risk factor for mortality in female patients with asthma. These results supported the hypothesis that depression might confer negative health effects on patients with asthma.

This study was the first to describe the impact of depression on long-term mortality in asthmatic patients among a nationally representative US population. Our findings underscored the importance of mental health screening for persons with asthma and the need for health professionals to relieve psychological distress in the management of asthma. As all we know, asthma was vulnerable to negative emotions and moods. According to previous studies, depressive disorder was common in asthma and was strongly associated with increased asthma symptom burden and worse health-related quality of life [18, 19]. Anxiety and depression were associated with poor asthma control, resulting in more visits to the doctor or emergency room among adults in the United States [12]. Furthermore, recent studies also found that control of depression would improve the management of asthma [20, 21]. In this study, we found that asthmatic patients with depression had a higher risk of all-cause mortality than those without depression and depression was an independent risk factor for all-cause mortality in females, which filled this critical knowledge gap that the impact of depression on long outcomes in asthma.

Several factors might explain why depression was associated with an increased risk of all-cause mortality in asthmatic patients. First, asthmatic patients with depressive symptoms were at high risk for poor adherence to asthma therapy, especially inhaled steroid regimens which were the most effective therapy available for patients with asthma [22]. Regular use of inhaled corticosteroids (ICS) not only reduced the risk of admission to the hospital, but also reduced the risk of morbidity and mortality for asthma [23]. Conversely, poor adherence to ICS caused by depression would increase the risk of death. Second, depression was associated with increased risk factors for mortality such as hypertension, cardiovascular disease, obesity, and type 2 diabetes [24–26]. Consistent with previous studies, we found that adults with asthma and depression were more likely to have obesity, hypertension, diabetes mellitus, coronary heart disease, congestive heart failure, stroke, emphysema, chronic bronchitis, and cancer compared with those with asthma only. Furthermore, depression itself might increase morbidity and mortality. Children with co-occurring asthma and depression were at increased risk for significantly elevated levels of inflammation and over time, which might be responsible for the development of additional chronic diseases and the increasement of asthma-related morbidity and mortality [27].

There were sex-related differences in the prevalence and prognosis of depression in this study. Female patients had a higher prevalence of depression and an increased risk of all-cause mortality compared with male patients. The exact mechanisms for these observed sex differences were not fully understood. Gene polymorphisms and female sex hormones might be important factors for sex-related disparities in asthma [28].

Our findings might have important implications for the management of asthma in clinic practice. Our study revealed that 14.16% of asthmatic patients had depression in the United States and the relative risk for death was increased 2.2‐fold in patients with co-occurring asthma and depression compared with those without depression. Depression was an identifiable, preventable, and treatable condition [29]. Therefore, clinical physicians should pay more attention to the early detection of depression in patients with asthma to ensure that they were appropriately managed, especially in female patients.

The current study had some limitations that need to be addressed. First, NHANES was a cross-sectional study, and PHQ-9 was evaluated at only one point in time, so adequate data on follow-up information of depression was not available in NHANES. It was acknowledged that the duration of depressive symptoms significantly affects patient prognosis; thus, measurement errors were inevitable in that information on PHQ-9 was only measured once. Second, this was an observational study and we were limited by the lack of information on the use of asthma medications and antidepressant medications. Hence, there was a risk of residual confounding (drug use) in this study. Despite these limitations, our study might provide important information regarding the necessity of routine depression screening in lowering the risk of mortality in asthmatic patients.

## Conclusion

In this study, we found that depressive disorder was common in asthmatic patients. Asthmatic patients with depression had a higher risk of all-cause mortality than those without depression and depression was an independent risk factor for mortality in female patients with asthma. Clinical physicians should pay more attention to the early detection of depression in patients with asthma to ensure that they were appropriately managed, which might improve asthma outcomes.

## Data Availability

Data used for this study are available on the NHANES website: https://wwwn.cdc.gov/nchs/nhanes/.

## Abbreviations

NHANES: The National Health and Nutrition Examination Survey
NDI: National death index
NCHS: The National Center for Health Statistics
CDC: The Centers for Disease Control and Prevention
ERB: Ethics review board
PHQ-9: The Patient Health Questionnaire-9
BMI: Body mass index
HR: Hazard ratio
CI: Confidence interval
ICS: Inhaled corticosteroids
